# An optimised protocol for the investigation of insulin signalling in a human cell culture model of adipogenesis

**DOI:** 10.1080/21623945.2023.2179339

**Published:** 2023-02-27

**Authors:** Jonathan M. Gamwell, Keanu Paphiti, Leanne Hodson, Fredrik Karpe, Katherine E. Pinnick, Marijana Todorčević

**Affiliations:** a Oxford Centre for Diabetes, Endocrinology and Metabolism, Radcliffe Department of Medicine, University of Oxford, Churchill Hospital, Headington, UK; b NIHR Oxford Biomedical Research Centre, OUH Foundation Trust, Oxford, UK

**Keywords:** Adipogenic cocktail, adipose stem cell, adipocyte, cell model, insulin signalling, glucose uptake

## Abstract

While there is no standardized protocol for the differentiation of human adipocytes in culture, common themes exist in the use of supra-physiological glucose and hormone concentrations, and an absence of exogenous fatty acids. These factors can have detrimental effects on some aspects of adipogenesis and adipocyte function. Here, we present methods for modifying the adipogenic differentiation protocol to overcome impaired glucose uptake and insulin signalling in human adipose-derived stem cell lines derived from the stromal vascular fraction of abdominal and gluteal subcutaneous adipose tissue. By reducing the length of exposure to adipogenic hormones, in combination with a physiological glucose concentration (5 mM), and the provision of exogenous fatty acids (reflecting typical dietary fatty acids), we were able to restore early insulin signalling events and glucose uptake, which were impaired by extended use of hormones and a high glucose concentration, respectively. Furthermore, the addition of exogenous fatty acids greatly increased the storage of triglycerides and removed the artificial demand to synthesize all fatty acids by *de novo* lipogenesis. Thus, modifying the adipogenic cocktail can enhance functional aspects of human adipocytes *in vitro* and is an important variable to consider prior to *in vitro* investigations into adipocyte biology.

## 1 Introduction

Obesity is closely associated with adipose tissue (AT) dysfunction and the development and progression of comorbidities, including Type 2 Diabetes, cardiovascular disease, non-alcoholic fatty liver disease, and some cancers ([1–3],). The growing burden of obesity-associated disease calls for a better understanding of adipose biology. To achieve this, reliably-performing human *in vitro* models are required to study processes which may influence disease pathogenesis, including adipocyte development, energy metabolism, and endocrine function.

Several human preadipocyte models have been established, including adipose-derived stem cells (ASCs) from the adipose stromal-vascular fraction ([4–7],), immortalized ASCs ([8–10],), and Simpson-Golabi-Behmel Syndrome (SGBS) cells [11]. However, there are limitations to each of these models and there remains great diversity in the adipogenic protocols used ([12,13],).

In our own work, culturing both primary and immortalized human ASCs, we have encountered functional issues, such as blunted insulin-stimulated glucose uptake, and this prompted us to re-examine protocols for ASC differentiation. Typically, ASCs are differentiated over 10–14 days in a defined adipogenic cocktail which is modified as differentiation progresses from an initial induction phase to a subsequent maintenance phase. Usually, the cocktail includes triiodothyronine (T3), dexamethasone (or other glucocorticoid) and insulin, while, for the induction phase only, (i.e. 0 to 4 days [14],), the cocktail is supplemented with 3-isobutyl-1-methylxanthine (IBMX) and a PPARγ agonist (e.g. troglitazone). The purpose of these components is to activate a cascade of adipogenic gene transcription, from the early expression of CCAAT/enhancer binding proteins, beta and delta (*CEBPB* and *CEBPD*), to later expression of the master regulator peroxisome proliferator activated receptor, gamma (*PPARG*) and *CEBPA* [15].

The concentrations of adipogenic additives vary significantly between research groups and cell lines, and are often supra-physiological ([13,16],). Chronic exposure to high concentrations of adipogenic hormones can alter cell function. For example, extended incubation with insulin impairs insulin signalling in mouse adipocytes [17], human myoblasts [18] and human umbilical vein endothelial cells [19]. Furthermore, there are successful examples in mouse adipocytes of IBMX and troglitazone being removed after 2, rather than 4 days [15], suggesting opportunities to reduce the use of hormones to improve function.

Supra-physiological concentrations (17.5–25 mM) of glucose are also widely used [7]. However, sustained high glucose concentrations may not be essential for successful adipogenesis [20] and have even been shown to impair insulin signalling in cultured mouse adipocytes [21] and rat skeletal muscle [22]. Furthermore, exogenous fatty acids (FAs) in the differentiation medium, representing the availability of dietary fats, are not commonly included in human adipogenic protocols, although they are standard in other models (fish, for example [10]. The intracellular triglyceride (TG) accumulation which accompanies adipogenesis is therefore largely dependent on the process of *de novo* lipogenesis (DNL), thus, putting artificial demands on lipid metabolism.

In this study, we explored modifications to the adipogenic differentiation protocol to restore insulin-stimulated glucose uptake by reducing the length of exposure to adipogenic hormones, adjusting glucose concentration (5 mM vs. 17.5 mM), and providing exogenous fatty acids at physiological concentrations (200 µM).

## 2 Methods

### 2.1 Cells

Immortalized (hTERT/HPV) abdominal and gluteal ASCs were generated from a healthy male donor and have been described previously^10^ [10].

### 2.2 Cell culture

Cells were cultured in DMEM/F12 Ham (Sigma, D6421) with 10% (v/v) foetal calf serum (Labtech), 100 U.mL^−1^penicillin/ 100 µg.mLstreptomycin (Invitrogen), 2 mM L-glutamine (Invitrogen) and 0.25 ng/ml fibroblast growth factor (R&D Systems).

### 2.3 Adipogenic differentiation

**Figure 1.**
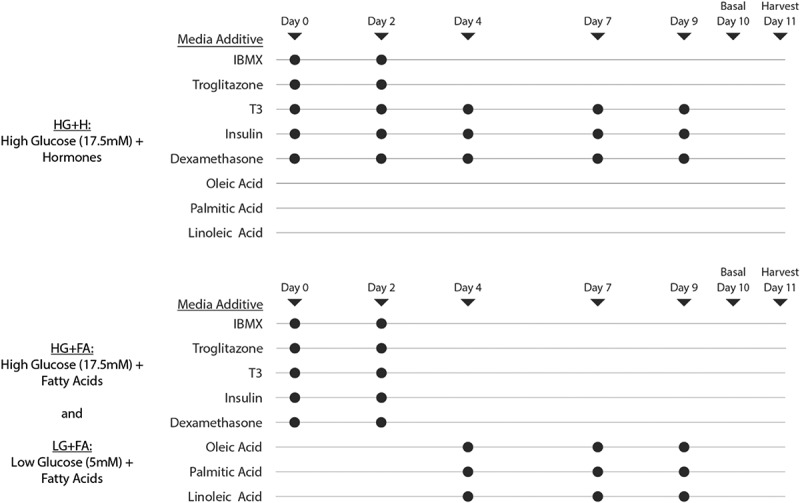
*Experimental design and composition of the modified adipogenic protocols*. On differentiation days 0 and 2, all three adipogenic protocols received hormones (100 nM insulin, 10 nM T3, 1 µM dexamethasone, 0.25 mM 3-isobutyl-1-methylxanthine and 4 µM troglitazone). Only the HG+H protocol continued to receive insulin, T3 and dexamethasone during media changes at days 4, 7 and 9. The HG+FA and LG+FA media were supplemented at days 4, 7 and 9 with a FA mixture (200 µM) containing the 3 major FAs found in the circulating NEFA pool in humans (45% oleate, 30%, palmitate and 25% linoleate) [36].

Cells were seeded at a density of 2 × 10^5^ cells per well onto 6-well plates and left to proliferate for 48 h until fully confluent before commencing differentiation. High glucose (17.5 mM) media was prepared using DMEM/F12 Ham solution (D6421) and low glucose (5 mM) media was prepared using glucose-free DMEM/F12 Ham solution (Biowest, L0091) with the addition of 5 mM glucose and the equivalent 15 mM HEPES. All media were supplemented with 100 U.mL^−1^ penicillin/100 µg.mL^−1^ streptomycin, 2 mM L-glutamine, 17 µM pantothenate, 33 µM biotin and 130 nM transferrin. Adipogenic hormones were added to relevant media (Figure 1) at the following concentrations: 100 nM insulin, 10 nM T3, 1 µM dexamethasone, 0.25 mM 3-isobutyl-1-methylxanthine and 4 µM troglitazone. FA stock solutions (10 mM) were prepared by dissolving sodium salts of oleate, palmitate and linoleate in 10% BSA prepared in DMEM/F12, at a ratio of 7:1/FA:BSA. FA stock concentrations were verified using the Randox Non Esterified Fatty Acids (NEFA) assay. The FA mixture added to relevant media comprised 45% oleate, 30%, palmitate and 25% linoleate and was applied at a final concentration of 200 µM, which is equivalent to the lower end of the plasma NEFA concentration range typically reported in humans ([23]).

For insulin signalling and glucose uptake experiments, differentiated cells were returned to basal DMEM/F12 medium at the appropriate glucose concentration, without hormones or FAs, for approximately 16 h before stimulation experiments commenced.

### 2.4 Insulin stimulation

Insulin stimulation was carried out on day 11. Insulin (25 nM) (Invitrogen) was added to each stimulated well while unstimulated wells were treated with vehicle (PBS). After 10 min incubation at room temperature, the wells were washed twice with PBS and cell lysis buffer added (cOmplete Lysis-M EDTA-free and PhosSTOP phosphatase inhibitor, Roche). After 3 min incubation on ice, the cells were scraped, transferred to sample tubes and stored at −80°C.

### 2.5 Western blotting

Cell lysates were sonicated (total 30 seconds) to ensure complete lysis and the protein content determined using the Bio-Rad DC assay. Western blots were carried out to detect the relative abundance of total-AKT, phospho-AKT S473 and alpha-tubulin. Bio-Rad Criterion stain-free, precast gels were loaded with 20 µg of protein. Gels were run in a tris-glycine-SDS running buffer in a Bio-Rad tank and a wet transfer with a trizma/ glycine buffer was used to transfer the separated proteins to a polyvinylidene-fluoride membrane. Membranes were blocked in BSA before an overnight incubation in the primary antibody (Cell Signalling 4058 (pAKT) (1:1000), 2920 (tAKT) (1:1000), 3021 (pIR) (1:800) & 3025 (tIR) (1:1000), Abcam 15246 (α-tubulin) (1:2000)) and a 1 h incubation in the secondary (31430 and 41360, both Invitrogen) before imaging on a Bio-Rad Chemidoc visualizer. Band intensities were measured using FIJI [24] and normalized against the alpha-tubulin intensities.

### 2.6 Glucose uptake assay

Glucose uptake was assayed on day 11 using a luminescent glucose uptake kit (Promega) in 96-well plates. Cells were washed with 100 μl of PBS, followed by incubation with 10 nM insulin for 30 min at 37°C in 5% CO_2_. To initiate glucose uptake, 50 μl of 2-Deoxy-D-Glucose (1 mM) in PBS was added to the cells. The samples were then processed as described in the manufacturer’s protocol. All assay steps were performed at room temperature. All data were acquired on a multimode plate reader instrument, with an integration time of 0.5 s.

### 2.7 Intracellular TG content

Cells were harvested for measurement of intracellular TG on day 10 of differentiation. Cells were scraped in a buffer containing 1% IGEPAL-630, 150 mM NaCl and 50 mM Tris HCl. Cell lysates were sonicated (total 30 seconds) to ensure complete lysis and the protein content determined as above. The samples were then heated at 95°C for 30 min, cooled and centrifuged at 13,000 x g for 10 min. The supernatant was collected for analysis on an iLAB 650 chemistry analyser using the Randox TAG assay kit. The total intracellular TG content was normalized to protein concentration.

### 2.8 High-content lipid imaging and analysis

Lipid: nuclei ratios were measured using a high-content imaging approach as described previously [25]. In brief, adipocytes in 96-well plates were fixed on day 11 of differentiation and fluorescently labelled for neutral lipids and nuclei using 3.8 μM BODIPY 493/503™ (Thermo Fisher Scientific, Cat. D3922) and 2 drops per ml NucBlue (Hoechst 33342, Thermo Fisher Scientific, R37605) respectively. Cells were imaged using a high-content imager (Cytation1, BioTek) and the fluorescent images analysed for the sum areas of lipid and nuclei signals in Gen5 software. The ratio was calculated by normalizing the lipid signal (BODIPY 493/503™) to nuclei signal (Hoechst 33342).

### 2.9 Fluorescent microscopy

Cells were differentiated on glass cover slips until day 14 before washing twice in PBS and fixing in 4% PFA for 30 min. Post fixing, cells were washed and stored in PBS at 4°C. Cells were permeabilised with saponin (0.1%) and stained with Hoechst 33342 (1:300), Oregon Green488 phalloidin (1:100, Invitrogen) and HCS LipidTOX Red Neutral Lipid Stain (1:200, Invitrogen) before mounting onto slides with VECTASHIELD mounting medium and imaging on a Nikon Eclipse CI microscope with Nikon DS-Fi3 camera. Single colour images were merged in FIJI [24] with minor adjustments to brightness and contrast.

### 2.10 Gene expression

Cells were washed in PBS and harvested in Tri-Reagent (Ambion, AM9738) by scraping. Total RNA was extracted and quantified (Nanodrop) before using 750 ng to synthesize cDNA (High Capacity cDNA Reverse Transcription Kit, Life Technologies, UK, 4368813). qPCR was performed on a 1/40 cDNA dilution using Taqman Assays-on-Demand (Applied Biosystems: *CEBPA*- Hs0029972_m1, *CEBPB*- Hs00942496_s1, *PPARG*- Hs01115729_m1, *ADIPOQ*- Hs00605917_m1, *LEP*- Hs00174877m1, *INSR*- Hs00961557_m1, *GLUT4*- Hs00168966_m1, *FASN*- Hs00188012_m1, *ELOVL6*- Hs00907564_m1, *SCD*- Hs00748952_m1, *DGAT2*- Hs01017541_m1, *PNPLA2*- Hs00386101_m1, *PLIN1*- Hs00160173_m1, *LIPE*- Hs00193510_m1) and Kapa Probe Fast Mastermix (Kapa Biosystems) on a QuantStudio 7 Flex (ABI). Triplicate reactions were performed in a final volume of 6 µL. Relative transcript expression was calculated using the ΔΔCt relative quantification method [26]. The ΔCt values of target genes were normalized to the ΔCt (geometric mean) of reference transcripts *18S, PGK1, PPIA* and *UBC*.

### 2.11 Statistics

Where indicated, Welch’s t-tests were used to test for differences between two conditions. Two-way ANOVAs and post-hoc Tukey tests were carried out to test for differences between multiple conditions (i.e. time, depot and media in the gene expression data). Significance was set at an alpha of 0.05 and the analysis was carried out using R v.4.1.1 [R Core 27] with figures generated with the ggplot2 package.

## 3 Results

### 3.1 Supra-physiological media glucose concentration impairs glucose uptake

Using high glucose and high hormone adipogenic differentiation media (HG+H protocol), we have previously observed impaired glucose uptake in human *in vitro* differentiated adipocytes (data not shown). We therefore set out to investigate whether insulin-stimulated glucose uptake could be restored using the modified adipogenic media and protocols we describe. All experiments were performed in parallel in paired abdominal and gluteal cells. Two modified adipogenic culture conditions were selected for detailed study (Figure 1); 1) HG+FA: high-glucose with FAs but without hormones after day 4, and 2) LG+FA: low-glucose with FAs but without hormones after day 4. Both protocols were compared to the standard HG+H protocol: high-glucose with adipogenic hormones but without FAs.

**Figure 2.**
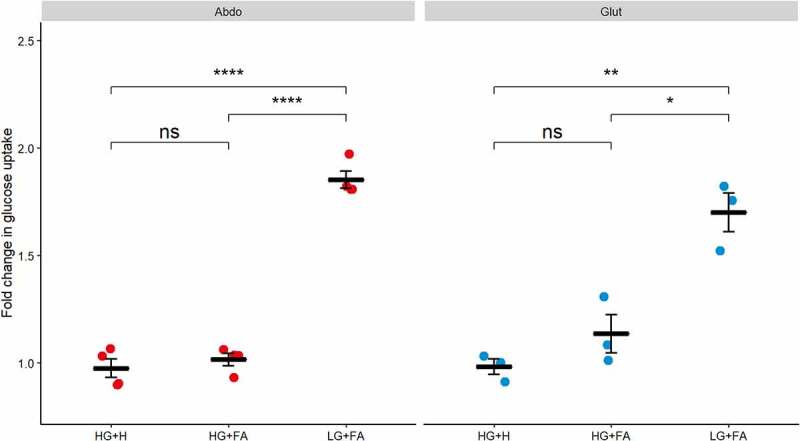
*Glucose uptake is impaired by high glucose*. Glucose uptake was measured after 30 min incubation with 10 nM insulin in immortalized abdominal (n = 4) and gluteal (n = 3) adipocytes following 10 days of differentiation in the three adipogenic media conditions. Horizontal bars represent means. Error bars represent SEM. HG+H, High Glucose (17.5 mM) plus hormones throughout differentiation; HG+FA, High Glucose plus FAs throughout; LG+FA, Low Glucose (5 mM) plus FAs throughout. Welch’s t-test tested for significance between basal and stimulated for each condition (*p < 0.05, **p < 0.01, ***p < 0.001, ****p < 0.0001).

Confirming our earlier observations, we found no significant increase in glucose uptake upon insulin stimulation in the HG+H media in both abdominal and gluteal cells (Figure 2). However, cells that were differentiated in the LG+FA media displayed an almost 2-fold increase in stimulated glucose uptake. By comparison, insulin-stimulated glucose uptake was not restored in either abdominal or gluteal cells differentiated in the HG+FA media (Figure 2).

### 3.2 Chronic exposure to adipogenic hormones impairs insulin signalling

**Figure 3.**
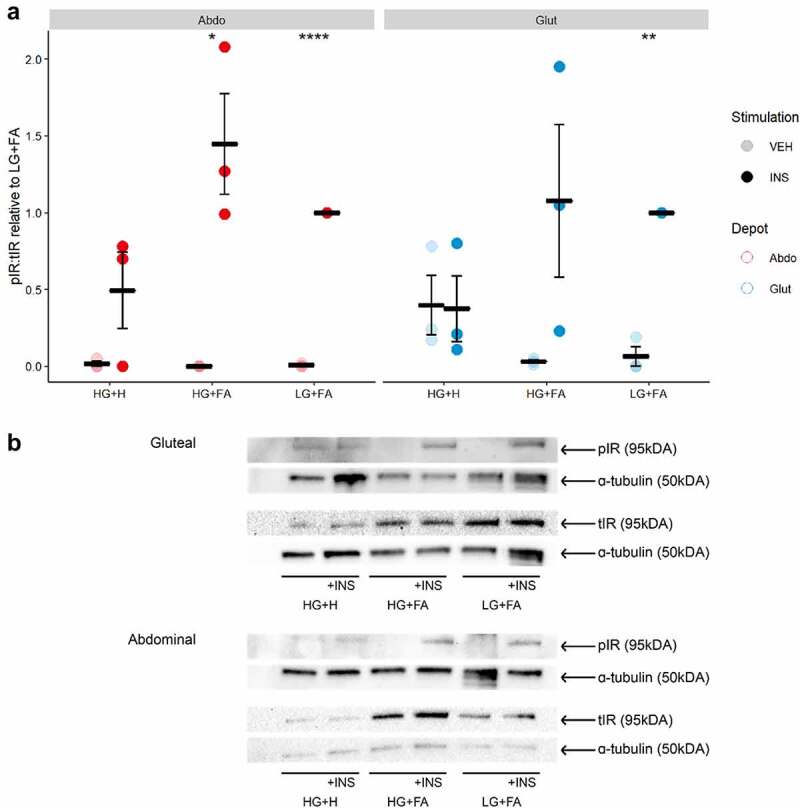
*Insulin receptor phosphorylation is impaired by chronic exposure to adipogenic hormones*. A: Ratio of phospho-IR (Tyr1131) to total IR after 10 min incubation with either Vehicle (PBS) or 25 nM Insulin in immortalized abdominal and gluteal adipocytes following 10 days of differentiation in the three adipogenic media conditions (n = 4). VEH, Vehicle; INS, Insulin; HG+H, High Glucose (17.5 mM) plus hormones throughout differentiation; HG+FA, High Glucose plus FAs throughout; LG+FA, Low Glucose (5 mM) plus FAs throughout. Welch’s t-test tested for significance between basal and stimulated for each condition (*p < 0.05, **p < 0.01, ***p < 0.001, ****p < 0.0001). B: Representative blot showing detection of pIR, IR and α-tubulin in abdominal and gluteal cells.

**Figure 4.**
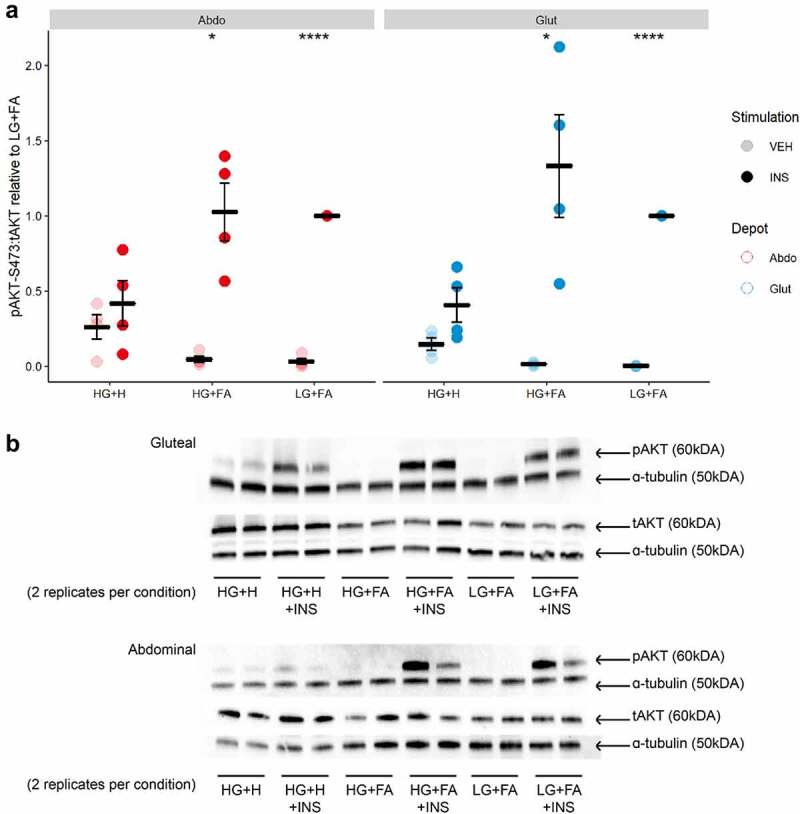
*Insulin signalling is restored by reducing the length of exposure to adipogenic hormones*. A: Ratio of phospho-AKT (Ser437) to total AKT after 10 min incubation with either Vehicle (PBS) or 25 nM Insulin in immortalized abdominal and gluteal adipocytes following 10 days of differentiation in the three adipogenic media conditions (n = 4). VEH, Vehicle; INS, Insulin; HG+H, High Glucose (17.5 mM) plus hormones throughout differentiation; HG+FA, High Glucose plus FAs throughout; LG+FA, Low Glucose (5 mM) plus FAs throughout. Welch’s t-test tested for significance between basal and stimulated for each condition (*p < 0.05, **p < 0.01, ***p < 0.001, ****p < 0.0001). B: Representative blot showing detection of pAKT, AKT and α-tubulin in abdominal and gluteal cells.

Next, we focused on events in the insulin signalling cascade by measuring insulin-induced phosphorylation of the insulin receptor (IR) at tyrosine 1131 (pIR ^tyr1131^) and AKT, a key downstream effector for insulin-mediated glucose uptake [28], at serine 473 (pAKT^ser473^). We observed a marked induction of both pIR^tyr1131^ and pAKT^ser473^ upon insulin-stimulation in cells differentiated in either LG+FA or HG+FA media (Figures 3 **and** Figure 4). This finding indicates a disparity between early events in the insulin signalling cascade (pIR^tyr1131^ and pAKT^ser473^) and the end measure of insulin-stimulated glucose uptake, with respect to HG+FA. By comparison, there was no significant induction of pIR^tyr1131^ or pAKT^ser473^ in cells differentiated in the HG+H media. Cells that were differentiated in HG+H media displayed elevated levels of pAKT^ser473^ in the vehicle (basal) condition (p = 0.0007); this contributed to, but did not fully explain, the impaired pAKT^ser473^ induction (Figure 4). Negligible pAKT^ser473^ was detected in the basal condition for LG+FA or HG+FA cells. Basal pIR^tyr1131^ was elevated, but not significantly, in the HG+H condition for gluteal cells.

### 3.3 Adipogenesis does not require the chronic use of adipogenic hormones

**Figure 5.**
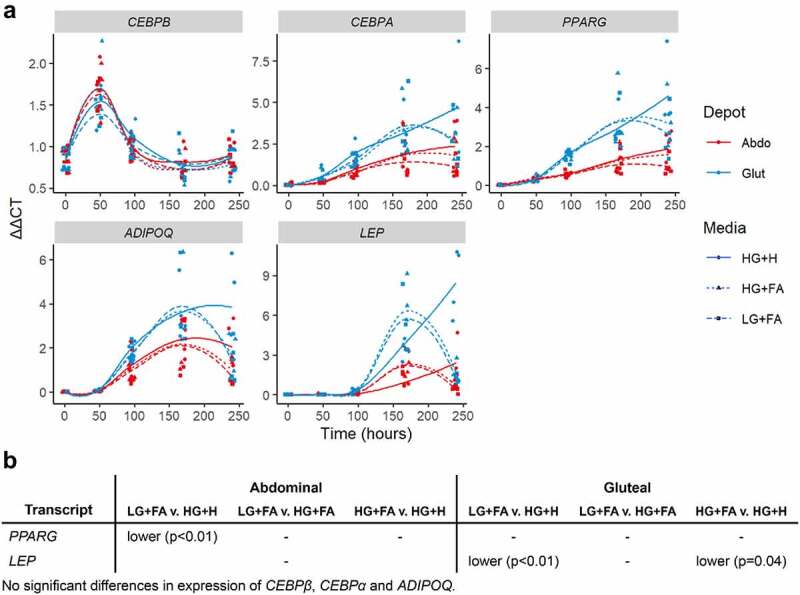
*Temporal expression profiles of adipogenic markers* A: Normalized gene expression (ΔΔCt) relative to housekeeper genes (*18S, PGK1, PPIA* and *UBC)* plotted at days 0, 2, 4, 7 and 10 with loess smoothed lines (n = 4). B: Table of significant differences based on two-way ANOVAs. HG+H, High Glucose (17.5 mM) plus Hormones throughout; HG+FA, High Glucose plus FAs throughout; LG+FA, Low Glucose (5 mM) plus FAs throughout.

Finally, to assess the effects of the modified protocols on adipogenic potential compared to the HG+H protocol, TG accumulation and gene expression were measured. The temporal expression of known adipogenic transcriptional regulators (*CEBPA, CEBPB* and *PPARG)* and mature adipocyte markers (*ADIPOQ* and *LEP*) showed few differences between conditions and indicated successful induction of adipogenesis by all protocols (Figure 5). The only exceptions were; higher *PPARG* in HG+H compared to LG+FA in the abdominal cells only, and higher *LEP* in HG+H compared to both of the modified protocols in gluteal cells only.

**Figure 6.**
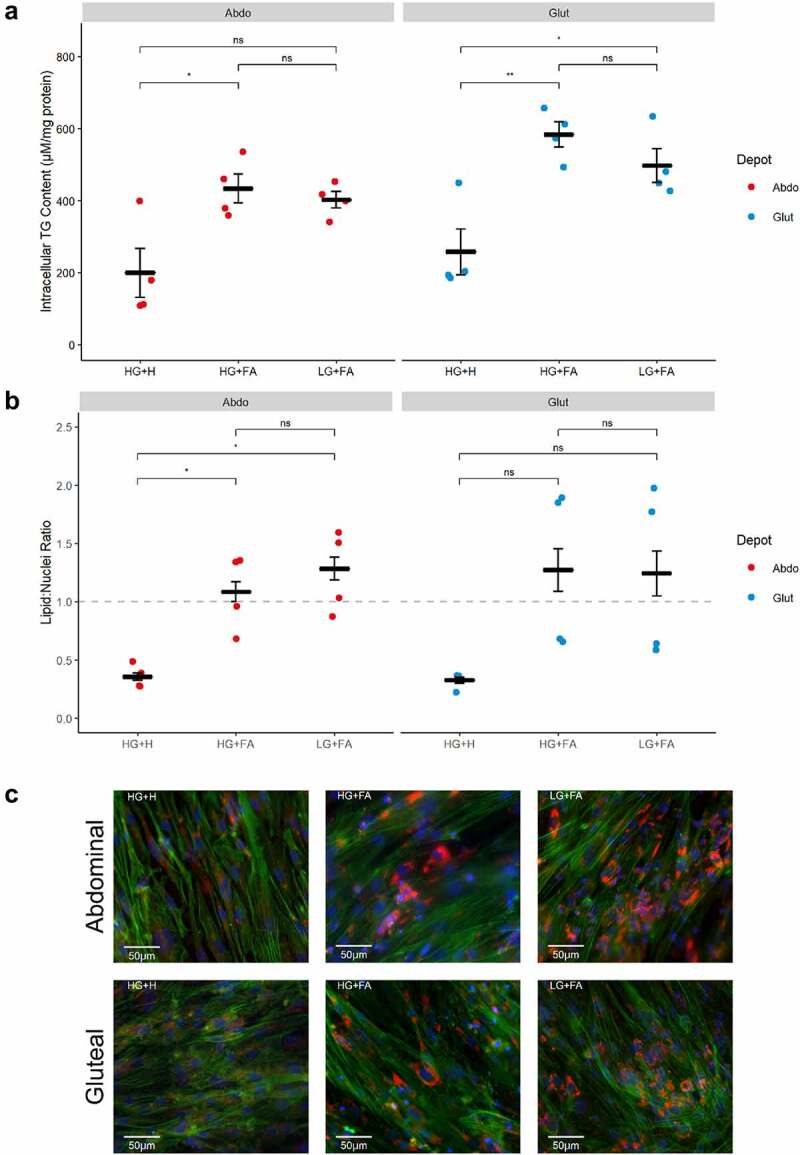
*Addition of exogenous FAs during adipogenesis promotes greater TG accumulation*. A: Normalized TG content of immortalized abdominal and gluteal adipocytes following 10 days of differentiation in the three adipogenic media conditions (n = 4). B: lipid: nuclei ratios in immortalized abdominal and gluteal adipocytes following 11 days of differentiation in the three adipogenic media conditions (n = 4). C: Representative fluorescent microscopy images of immortalized abdominal and gluteal adipocytes following 14 days of differentiation in the three adipogenic media conditions. Neutral lipids in red, nuclei in blue and cytoskeleton in green. Horizontal bars represent means. Error bars represent SEM. TG, triglyceride; HG+H, High Glucose (17.5 mM) plus Hormones throughout; HG+FA, High Glucose plus FAs throughout; LG+FA, Low Glucose (5 mM) plus FAs throughout. Welch’s t-test tested for significance between protocols for each condition (*p < 0.05, **p < 0.01).

All cells accumulated TG by day 11, indicative of adipogenic differentiation (Figure 6a). Intracellular TG content was significantly higher in cells cultured with the modified protocols (HG+FA or LG+FA) compared to HG+H (p < 0.001), with no further differences between the two modified protocols (p = 0.98).

High-content imaging identified higher lipid: nuclei ratios in the modified protocols compared to the HG+H condition in abdominal cells (p < 0.05) with a similar trend in gluteal cells (Figure 6b). The higher ratios were driven by a strong increase in lipid signal, with larger lipid droplets observed in differentiated cells (Figure 6c). However, even under the modified protocols it was clear that not all cells underwent adipogenic differentiation owing to the mixed background of these cell lines.

**Figure 7.**
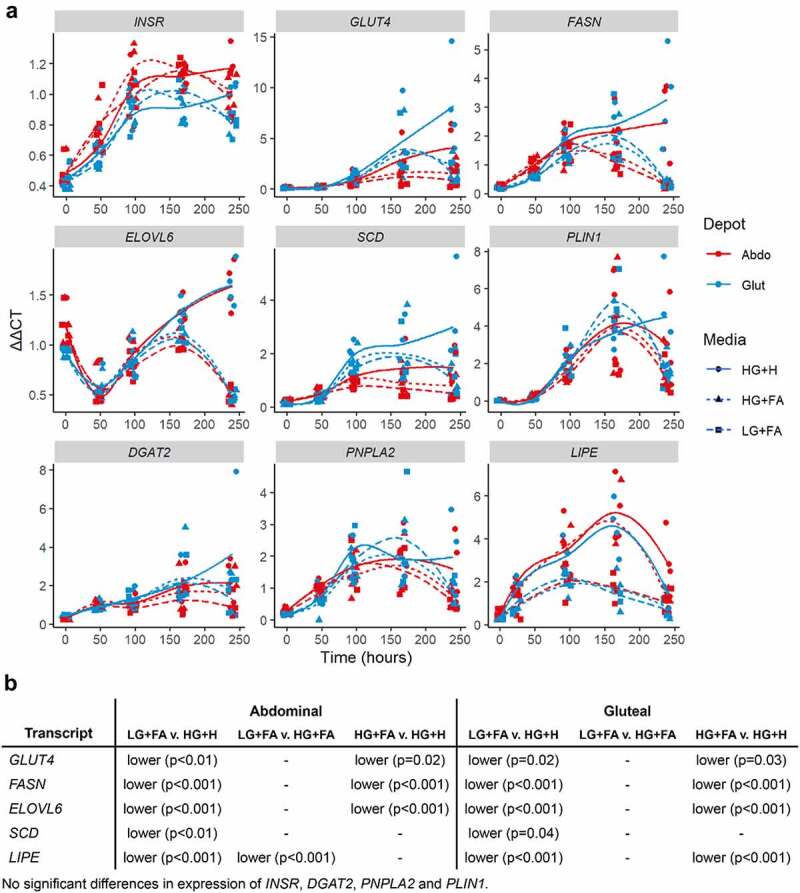
*Temporal expression profiles of genes associated with de novo lipogenesis and lipid handling*. A: Normalized gene expression (ΔΔCt) relative to housekeeper genes (*18S, PGK1, PPIA* and *UBC)* plotted at days 0, 2, 4, 7 and 10 with loess smoothed lines (n = 4). B: Table of significant differences based on two-way ANOVAs. HG+H, High Glucose (17.5 mM) plus Hormones throughout; HG+FA, High Glucose plus FAs throughout; LG+FA, Low Glucose (5 mM) plus FAs throughout.

To further assess differences in lipid storage and handling between HG+H and the modified protocols, a panel of glucose and lipid metabolism genes were run. Expression levels of genes relating to DNL were found to be elevated in the HG+H (p < 0.05) condition (*GLUT4, FASN, ELOVL6, SCD*) (Figure 7) while, despite the greater amount of TG in the modified protocols, genes relating to lipid management and handling tended to be similarly expressed in all conditions with the exception of *LIPE* which was lower in LG+FA than other conditions in the abdominal cells and higher in HG+H than the other conditions in gluteal cells.

## 4 Discussion

In this study, we report that adjusting the composition of the adipogenic differentiation cocktail influences adipocyte function, and we propose that hormonal and nutritional components of the cocktail should be carefully considered when planning *in vitro* experimental work in ASCs, as these factors may influence the study outcomes. This is in keeping with the recommendations outlined by Lagziel et al. [29], who argue that common culture media often contain un-physiological concentrations of metabolites, resulting in negative effects on key metabolic processes.

Our goal was not to fine-tune every component of the cocktail, but rather to see whether by adjusting the media composition we could overcome technical challenges previously encountered when assessing glucose uptake and insulin signalling in ASCs differentiated *in vitro*, whilst still achieving successful adipogenic differentiation. Contrary to established protocols [16], our results indicate that under certain experimental conditions, adipogenic hormones are not required throughout the duration of differentiation for successful adipogenesis. In fact, the absence of hormones (T3, dexamethasone and insulin) during the maintenance phase, combined with the addition of FAs, reversed the insulin insensitivity observed in adipocytes treated with HG+H. Our findings are consistent with those from murine models; Nelson et al. showed that insulin sensitivity (measured by phosphorylation of AKT) was intact when insulin was removed after the induction phase but lost if the cells were re-exposed to insulin [17].

While the failure of IR and AKT phosphorylation early in the insulin-stimulated glucose uptake pathway was independent of glucose concentration, we also found that glucose uptake itself was impaired by high glucose and this was independent of the extended presence of hormones. The media concentration of glucose varies between published differentiation protocols and is often higher than physiological (usually 17.5–25 mM but on at least one occasion, 70 mM [7]. Supra-physiological concentrations of glucose affect glucose uptake in cultured mouse adipocytes [21] and our data confirms this in human cells. Furthermore, the reduction in glucose concentration from 17.5 to 5 mM had no discernible effect on adipogenesis (as assessed by TG accumulation and gene expression) showing that it is not necessary to utilize high glucose concentrations throughout differentiation. This is in agreement with Jackson et al. [20] who found that a high concentration of glucose was only required for the first three days to ensure differentiation in murine adipocytes.

FAs are not commonly included in human adipogenic protocols, which is questionable, since the primary role of adipocytes is to take up, store and release dietary FAs, rather than to synthesize them *de novo*. Whole-body DNL is estimated to account for, at most, 20% of palmitate stored in human AT, with adipocytes contributing only a fraction of this [30]. In our HG+H protocol, genes involved in DNL were expressed at elevated levels reflecting the dependence of cultured ASCs on DNL for lipid synthesis and storage when exogenous FAs are not provided in the culture media. This could have detrimental effects on adipocyte function as it results in a cellular FA composition which is deficient in the essential FAs (18:2 *n-6* and 18:3 *n-6*) and their products, these are needed for vital processes including bioactive lipid signalling, maintenance of membrane structure, the inflammatory response, and modulating gene expression [31]. Here, supplementation of the media with FAs (including 18:2 *n-6)* led to greater TG accumulation and larger lipid droplet size, thus, as *in vivo*, there is not a reliance on, or a limitation from, DNL, which is an important consideration when studying adipocyte FA metabolism. Several adipocyte markers displayed altered gene expression levels in some of the modified protocol conditions compared to the standard HG+G protocol (*PPARG, LEP, LIPE*). Glucocorticoids, insulin and FAs [32–34], are known modulators of adipocyte gene expression and function. For example, *LIPE* gene expression is known to be upregulated in the presence of dexamethasone [32], therefore it is not surprising that some changes in gene expression were detected when hormones were removed and FAs added. However, these changes in gene expression were not related to detrimental effects on adipogenic capacity, glucose uptake or insulin signalling.

ASCs from different AT depots display developmental and functional differences which are retained in culture *ex vivo* [35]. Differences in insulin signalling between abdominal and gluteal adipocytes have not previously been reported. Here, we show that high glucose and hormones negatively impact on glucose uptake and insulin signalling in cells from both abdominal and gluteal depots to a similar degree, and that restoration of insulin sensitivity in response to our modified protocols is comparable between both depots.

Overall, we have shown that different components of the adipogenic media can have detrimental effects on adipocyte function when at supra-physiological levels and these can be avoided by modifying the differentiation protocol. We highlight the importance of understanding the elements of the adipogenic cocktail and the potential for optimizing the differentiation protocol for the investigation of FA metabolism and insulin signalling for *in vitro* models of adipogenesis.

## Data Availability

The data that support the findings of this study are available from the corresponding author, K.E.P., upon reasonable request.
